# Recontacting in medical genetics: the implications of a broadening knowledge base

**DOI:** 10.1007/s00439-021-02353-5

**Published:** 2021-08-30

**Authors:** Shane Doheny

**Affiliations:** Cardiff University Institute of Cancer and Genetics, Cardiff, SGM UK

## Abstract

The practice of recontacting patients has a long history in medicine but emerged as an issue in genetics as the rapid expansion of knowledge and of testing capacity raised questions about whether, when and how to recontact patients. Until recently, the debate on recontacting has focussed on theoretical concerns of experts. The publication of empirical research into the views of patients, clinicians, laboratories and services in a number of countries has changed this. These studies have filled out, and altered our view of, this issue. Whereas debates on the duty to recontact have explored all aspects of recontact practice, recent contributions have been developing a more nuanced view of recontacting. The result is a narrowing of the scope of the duty, so that a norm on recontacting focuses on the practice of reaching out to discharged patients. This brings into focus the importance of the consent conversation, the resource implications of this duty, and the role of the patient in recontacting.

## Introduction

In medical genetics, questions about recontacting emerge in the wake of advances in knowledge. These include new interpretations of existing genes, new or more accurate tests, or new understandings of the effect that combinations of genes have on diseases and disease outcomes. This new knowledge affects practice as it generates expectations that genetics services recontact patients with updates. Ideally, a ‘duty to recontact’ would provide an uncontroversial norm guiding action whenever a professional in a genetics service considers recontacting a patient. This putative norm would provide guidance on the kinds of information that warrant a recontact, how to weigh the interests of patients, their families, professionals and healthcare providers, provide a steer on the importance of time, and indicate how to conduct the recontact. Each element creates problems. For instance, how long after a patient is discharged is it acceptable to recontact them? What information warrants a recontact? What should professionals do if the patient has said they do not wish to be recontacted? These problems have engaged practitioners, researchers and scholars in debates over the past three decades. What began as a theoretical debate has expanded to include the voices and views of practitioners and patients. This has led to refinements in what we mean by the duty to recontact, but has also changed the terms of the debate, and raised concerns with the practical and resource implications of practicing recontact.

## Early debates on the ‘duty’ to recontact

Early ideas on recontacting emerged from debates among clinicians and ethicists. In their systematic review of this debate, Otten et al. (2014) mapped its features and observed how it was dominated by clinicians and ethicists in the 1990s and 2000s, who conducted their debates initially in the context of targeted genetic tests and subsequently in relation to information gleaned through whole genome sequencing. This dominance was reflected in the use of general ethical principles to consider the effect that recontacting might have on patients, but with little input from patients (with the exception of Fitzpatrick et al. 1999). They focussed on liability, the potential effect that a legal backing to recontact may have (Otten et al. 2014: 671) and the sparsity of empirical evidence and formal guidelines for recontact. The conclusion Otten et al. (2014) reached was that they could find no general legal basis for recontacting, though it was “often” considered desirable. They called for a shift “from the current discussion of whether there is a general duty to recontact in clinical genetics and focussing on the question of in which specific situations recontacting might be regarded as a good standard” (2014: 667).

Since 2014, the discourse shifted as studies have been conducted that gather the views of various of the groups affected (Carrieri et al. 2016; Dheensa et al. 2017; Doheny et al. 2018; Vears 2018a, b, Mitchell et al. 2020). The theory focussed debate has expanded to include voices of interested parties, and these voices have addressed many of the practical issues in recontacting.

## Organising recontacting: who triggers the recontact?

The question of what triggers a recontact has been the concern of many involved in recontacting in genetics. The specific trigger may be a reinterpretation of a test result, a new test or some newly discovered clinical utility. Thus, decisions on who triggers a recontact shapes how recontacting is absorbed into organisational roles and responsibilities.

Both Carrieri et al (2017a, b) and Sirchia et al (2018) report on surveys of genetics services (one UK focussed, the other European) and suggest that most services consider that new information that would impact on the clinical management of the patient or their family is the most important trigger. However, they also note a preference for recontacting a small number of patients, therefore the scale of recontact and the additional demand it may create was a consideration. Nevertheless, these are primarily surveys of the views of service managers. The question of who decides to make a recontact raises questions about the role of clinicians, laboratory staff, and of the patient.

## The clinician

In an analysis of qualitative interviews with clinicians and counsellors in genetics, we Doheny et al. (2018) explored clinician’s views on responsibilities to recontact. Doheny et al. (2018) found that clinicians held significantly different views on their roles in relation to recontacting. Some felt all clinicians had a responsibility to review all of their patients with a variant of uncertain significance (VUS) result annually, to see if there was a need to recontact these patients. Others drew attention to their personal limitations (the limitation of memory, the realities of their workload, or the implications of moving to another post or of retiring) to emphasise the limits to their responsibilities. Concluding this study, we (2018: 204) drew attention to how this contrasting sense of responsibility creates risks for policy makers insofar as a prescriptive policy framework may bolster the sense of responsibility of many professionals while weakening the commitment of those who see such a recontact policy as onerous.

## The laboratory

If the responsibility to trigger a recontact is not to be located with clinicians, then it may be located with either laboratories or patients. A working group convened to reflect on the points laboratories need to consider in relation to NGS technologies observed that there is no duty for laboratories to reanalyse sequence data in light of additions to variant databases, and laboratories are only expected to provide the initial analysis (Vears et al. 2018a). Nor is there an expectation that laboratories reanalyse data (Vears et al. 2018a). However, the working group took a different view with respect to the ‘reinterpretation’ of existing data where a variant is reclassified from benign to pathogenic, or pathogenic to benign. In these cases, variants are being “reinterpreted” and “it is good clinical practice for the laboratory to reissue a report to the referring clinician so they can attempt to communicate this information to the patient” (Vears et al. 2018a: 42). El Mecky et al. (2019) add empirical data gathered using online focus groups conducted with laboratory geneticists in the Netherlands. El Mecky et al.’s (2019) participants reinterpreted data in response to requests from patients and clinicians. They felt that active reinterpretation by the laboratory was not feasible. However, while El Mecky et al’s (2019) participants communicated any new information they found to patients or clinicians upon request, they did not have a default procedure to communicate new information to clinicians when a variant is reclassified. Instead, laboratories communicated reclassifications they considered to be relevant (i.e., a reclassification to likely pathogenic, or a downgrade from pathogenic to VUS) and did not report changes from benign to likely benign as this would not change medical management.

Interestingly, the laboratory scientists expressed anxieties about the potential recontact that may result from reinterpretation. In practice, laboratory scientists would reinterpret a variation during the analysis of a new patient, but while this reinterpretation may affect the interpretation of variations in past patients, these others had not consented to the reinterpretation of their variations. The scientists were concerned that an ensuing recontact may involve a patient who no longer wanted to be contacted, or had not realised the durability of their initial consent to reinterpretation and recontact (El Mecky et al. 2019: 4).

## The patient

If the organisational locus of recontacting is not to be situated with the lab, or with clinicians, then perhaps it could be located with patients? This is investigated by Dheensa et al. (2017) in an analysis of interviews with patients who had been recontacted. The interviewees in Dheensa et al.’s (2017) study felt that patients could not be expected to instigate a recontact due to their lack of insight on when to recontact, and their inability to ensure a recontact led to a clinic appointment. The patients also pointed out that the option to recontact might cause patients to ‘dwell’ on an otherwise past illness. They felt professionals had a duty to recontact because they had better access to, and understanding of, the relevant information. But they “did not argue that the health service should be solely responsible for recontacting but that recontacting should be a “*joint venture*” (P37) between patients/parents and professionals” (2017: 406). Their case for a ‘joint venture’ was not based on protecting patient autonomy, but was seen as a practically feasible way of managing recontacting in the environment of a healthcare system with limited resources. Following from this pragmatic view is a need for a mechanism that would make a duty to recontact possible in the first place. For these patients, an information sharing system that enables recontact to happen needs to be in place to make it possible to speak of a ‘duty’ to recontact. Finally, some of these patients recognised that a joint system would shift the concerns about responsibility to a different set of issues. No longer would the concern be about whether or how to re-establish contact but about those patients who did not respond to their clinicians recontacting them, so that they were unable to make use of the new information. As Dheensa et al. (2017) note, any information sharing system would encounter problems around data protection, the kinds of information that may be stored, the threshold to trigger a recontact, and the potential barriers of social class and education on patient use of such a system (see also, Bombard and Mighton 2018).

Situating triggering mechanisms for a recontact with any of these three actors creates issues in each case. Research data on the views of clinical and laboratory staff as well as patients contributes to this debate by extending it to include the concerns of these actors. One question that is not addressed here concerns the role of voluntary or charitable sector organisations. With the development of novel forms of biological citizenship, voluntary sector interest groups have a role in advancing the interests of particular patient groups both by supporting, and in some cases funding, research into the causes and treatments for these conditions (Rose 2007). Such organisations are well placed to take a key role in recontacting, but the voice of such organisations is so far absent from this literature.

## Consent to recontact

The practice of professionals getting back in touch with a patient can take a variety of forms. Clinicians, counsellors or laboratory technicians may recontact patients, whom they may or may not have previously met, to discuss new information. If the person doing the recontact has met the patient before, they may remember the patient and have a sense of how the patient might respond to a recontact. If they have not met the patient before, they rely on the information on the consent form to decide how to proceed. In their survey of the recontacting practices of genetic services in the UK, Carrieri et al (2016) found that a slim majority of providers ask patients about their recontacting preferences. Around a third (six of 20 providers) made a systematic recording of preferences with another third doing so occasionally and the remainder not recording preferences. Nevertheless, the consent form did not appear to matter a great deal to providers as a larger majority (14 of 20) reported their willingness to recontact patients even if patients had indicated they did not want to be recontacted. This willingness to overrule patients’ earlier, hypothetical preferences could be justified where clinicians felt that the new information had clinical implications.

By analysing the content of consent forms, Vears et al (2018b) examine the reasons that patients are given that explain why they might be recontacted in the future and map the ways in which reinterpretation and recontact is mentioned on consent forms. On this analysis, recontact may arise in these conversations:

- in relation to the *reinterpretation* of results by pointing to the possibility that new information may become available in the future or the variant becoming reclassified.
- in terms of the initiation of a reinterpretation, for instance, where a patient, clinician or laboratory get in touch to discuss or to request a new interpretation
- forms may or may not mention recontact in general terms, or
- (d) forms may indicate who may carry out a recontact or has a role in initiating a recontact (summarising table 4 of Vears et al. 2018b: 1747)

Consent forms provide an organisation and structure to the consent conversation that clinicians and counsellors have with patients, so the extent to which the form guides conversations to make patients aware of the complex reasons that their information may be managed and reviewed is of vital importance. Vears et al. (2018b), point to a range of shortcomings in how forms navigate these difficulties, while mindful of the complex nature of the problem these forms are addressing. The first problem Vears et al (2018b) raise is about the reinterpretation of data. As noted above, laboratories are not duty bound to reanalyse the data of individual patients when changes are made in variant databases, but only a third of the consent forms included in Vears et al’s (2018b) analysis made it clear that reinterpretation may take place, while a large proportion did not mention this possibility. Less than half of these consent forms mentioned recontact for clinical purposes and, of those that did, Vears et al (2018b) noted “considerable variation” on who was responsible for initiating recontact.

### (Re-)Contacting patients

This leads us to the practicalities of how a service may recontact a patient. There is little analysis of how services actually do get back to patients. Beunders et al. (2018) compared the use of the phone with three different letters sent to patients, and noted that patients contacted by phone were far more likely to make an appointment with their clinician following a recontact, thus underlying the importance of considering the method used by healthcare professionals to recontact. Doheny et al. (2018: 16, 17) provide some insight on recontact modalities in the context of an analysis of how clinicians describe their responsibilities. This was an analysis that focussed on clinical genetics, but is suggestive of the nuance that is involved in the enactment of recontact. On this analysis each patient needs to be managed based on their circumstances—work, family relationships, capacity to understand and willingness to engage with the information.

Finally, there have been limited studies on the effect that the receipt of a recontact request may have on patients. Dheensa et al (2017) draw attention to how patients they interviewed shared a concern that a recontact may cause distress for patients. Drawing on the same study, Carrieri et al (2017a, b) describe how patients are supportive of recontacting but are sceptical about whether the NHS will have the necessary resources. Moreover, Carrieri et al. point to the complex emotions that some patients experience as a result of being recontacted, underlying the need for discussion on the possibility of recontacting in the initial consent conversation between HCPs and patients. These topics are underexplored in the research literature.

### Policy and guidance on recontacting

In their earliest guidance on recontacting, the American College of Medical Genetics (ACMG) (Hirschhorn et al. 1999) considered that the primary care physician played a key role in mediating between the patient and medical genetics services. They reasoned that GPs know the family and their history, whereas genetic services may lose contact with a family. The guidance considered any lapse of time as irrelevant, but changes in family circumstances and advances in knowledge may not be. In this framework, the patient plays a role by prompting their GP to reconsider genetic information. This placed responsibility to recontact with the GP as the professional best placed to assess the need to recontact in light of the patient’s wishes despite the fact that the GPs may have limited insight into developments in genetics. This raises difficulties for genetics services wishing to recontact patients who did not want their GP aware of their genetics consultations. Overall, this guidance is more suited to situations where the test itself is important, and recontact is not driven by the discovery or new knowledge. It renders the genetics service passive, as it waits for a GP to call to request an update and provides no guidance for a genetics service who discover new genetic information that warrants recontacting a patient.

In 2007, the ACMG (Richards et al. 2007) revised its guidance on recontacting in the context of novel sequence variations, and recommended that the testing laboratory contact physicians of patients where new information emerges. This places some responsibility on the clinician to assess the need to recontact, and on laboratories to retain some review activity on existing rare variants. In its most recent guidance, the ACMG (David et al. 2018) sees laboratories, patients, and physicians as sharing responsibility for communication where the clinical meaning of genomic data changes. This guidance positions the ‘ordering provider’ as the primary coordinator of care, but ultimate responsibility to initiate a recontact is left with the patient, raising the problem of the inverse care law (Tudor Hart 1971). The main line of argument here is that the complexity of genetic and genomic data is such that the interpretation of this data may change over time to become more accurate or more complete. This can be to the benefit of the patient, thereby creating a ‘duty to reinterpret’.

More recent guidance tackles recontact in the genomic context. A report by the Joint Committee on Genomics in Medicine in the UK (JCGM 2019) focussed on issues of consent and confidentiality in genomics. This focussed on the reinterpretation of genomic data, where information stored on a patient’s genomic data may be interpreted in light of new clinically relevant information. On this guidance, the responsibility to initiate a reinterpretation of patient data does not rest with any one actor. Emphasis is placed on the consent conversation as an opportunity to inform patients on the roles and expectations of those involved. Patients are made aware that they may be recontacted at any time in the future, and that the patient may also recontact genetics services. This situation of an ongoing but non-specific responsibility to initiate recontact on both the laboratory, the clinic and on patients was situated in the context of a system that lacks a consistent framework and with the “hope that this will be technically and logistically feasible in the future” (2019: 18). But as the JCGM (2019: 32) points out, by giving patients responsibility to revisit their genetic advice, laboratory scientists and clinicians need to clarify their responsibilities when recontacted by patients.

At the same time, the ESHG published recommendations co-authored with the researchers involved in the UK study on recontacting (Carrieri et al. 2019). Drawing on empirical research as well as ethical and clinical opinion, these authors set out twelve recommendations for the management of recontact. This guidance focuses on the process of re-establishing contact with a patient, providing indications on the information that may justify a recontact, the need to consider the capacity of the healthcare system and the resources required, and the sharing of responsibility for initiating a recontact.

## Recontact and the law

While guidance structures and orients the judgement exercised by healthcare professionals as they make their decisions about recontacting, legal discourses have focussed on the core element that needs to be evident in professional deliberations. Legal scholars have pointed out that the duty to recontact may have legal backing from a number of sources. Ploem et al. (2018) note that at the European level, articulated in the European Convention of Human Rights and by the Council of Europe, patients and citizens have rights to access health information that has been gathered about them, and to the information about their health gleaned from a test. However, as Ploem et al. (2018) also note, it is not clear how this right might apply in genetics where new information emerges from reanalysis and reinterpretation of existing data (2018: 545). Such a duty may be supported by the right that patients have “to receive all the health information that is available to them” (Ploem et al. 2018). At a national level, Ploem et al. (2018) discuss the responsibilities of healthcare professionals to provide post-treatment care where the ongoing relationship between the clinician and the patient ceases. The difficulty, however, arises from the practicalities of managing voluminous and difficult data for purposes of reinterpretation which places limits on any right to be recontacted (2018: 547). Overall, legal scholars argue that a legal duty to recontact is a desirable but unrealistic goal, given the existing resource, technological and organisational constraints (Ploem et al. 2018: 553). However, Ploem et al. add that healthcare professionals can still be expected to do the best that can reasonably be expected of them, and that technological advancements are likely to lead to an expansion of the legal duty to recontact (2018: 553).

A separate question centres on what is expected of clinicians and laboratory scientists who are considering whether or how to recontact patients in any given circumstance. These issues take us back to questions about the duty of confidentiality and the duty of care owed by healthcare professionals as they deliberate a decision to recontact. The recent conclusion of the *ABC* (see Dove et al. 2018; de Paor 2018) case has led to the clarification that healthcare professionals do have limited duties to disclose details of relevant genetic risk to family members (Dove et al. 2018; de Paor 2018; Mitchell 2020). However, as Mitchell (2020) notes, the legally enforceable duty “is more limited than existing professional guidance” issued by the BMA or the JCGM (2019) as professional guidelines already direct professionals to balance issues of consent with concern to reduce the risk of harm. The *ABC* case has instead clarified the need for documenting the decision-making process.

What follows from the professional guidance and law around recontacting is the need for insight into how professional bodies may prompt healthcare professionals to develop and evidence their considerations as they deliberate recontacting a patient.

## Sharpening the duty to recontact

While adding breadth and depth to the discourse on recontact, the research contributions enable us to sharpen what we mean by recontact, and to focus this duty in certain circumstances and roles. Interestingly, Applebaum et al. (2020: 634) view the duty to recontact as the final element of a more general ‘duty to reinterpret’. For Applebaum et al. (2020) this duty contains four elements—the storage of data, initiation of reinterpretation, reinterpretation of the data, and recontact of the patient. While they argue that the general duty to reinterpret is well supported, particularly by the principles of patient autonomy, beneficence and non-maleficence, they recognise the arguments against this duty that flow from its practical and ethical implications. The main line of argument here is that the complexity of genetic and genomic data is such that the interpretation of this data may change over time to become more accurate or more complete. This can be to the benefit of the patient, thereby creating a ‘duty to reinterpret’. Consequently, the production and interpretation of genetic/genomic data gives rise to duties to store data to make reinterpretation possible; to provide an actor or actors with the power to trigger a reinterpretation of data; a process of reanalysing data and, finally, a process for recontacting a patient where pertinent new information comes to light. On this final point, Applebaum et al. (2020) contend that either the laboratory or the ordering clinician should recontact the patient, depending on how adequate counselling could be provided. By locating the duty to recontact as an ethical obligation that flows from a more general duty to reinterpret, Applebaum et al. (2020) separate out some of its elements and fill out the moral principles. In effect, the duty to get back in touch gains its proper place as an ethical obligation that flows from the production of new knowledge. Many of the issues that generated problems for recontact—like the question of time and the kind of information that warrants a recontact—are separated out as issues in the management of knowledge. The moral norms that concern recontact become those concerning who will conduct the recontact, how they go about it, and what to do where patients do not respond.

Applebaum et al.’s (2020) intervention introduces an interesting change to how we conceptualise recontact. Overall, the duty to reinterpret or to recontact emerges in the wake of advances in knowledge. These are the ethical implications of such advances, but the advance itself is indifferent to its own ethical implications. By separating the duty to recontact from the duty to reinterpret, Applebaum et al. (2020) separate ethical problems of management and storage of knowledge from ethical problems that arise as the clinical service reaches out to the patient. This separation echoes the sociological treatment of Doheny et al. (2018) where we delineated the responsibilities inherent in the duty to recontact into responsibilities operative in systems and those operative in the lifeworld. Thus, the duty to reinterpret focuses on internal systems within medical genetics, and the duty to recontact focuses on the bridging of clinical systems with the family and lifeworld. This important distinction relieves the duty to recontact of much work around the evaluation and management of knowledge, instead focussing on the issues that had originally concerned it, i.e. the management of communication between the medical genetic system and a discharged patient. Taken together, the contributions of Applebaum et al. (2020) and Doheny et al (2018) begin to break up the duty to recontact so that issues like the knowledge that triggers a recontact, the lapse of time between contacts, or the management and storage of information leading to a recontact are separated. The issue for recontacting is then narrowed to those tasks and issues that emerge in the process of reaching out to a patient. This implies that a norm on recontact focuses on the modality and ethics involved in the process of making contact, and the management of a conversation with a patient who may have forgotten about genetics or is uninterested in being recontacted.

## Summary

Debates on recontacting conducted among experts have, in the past decade, expanded to incorporate the views and experiences of those affected, providing grounds for nuanced guidance for practitioners. But gaps remain in the research literature. Little is known about the methods of recontacting most likely to result in clinic appointments, and virtually nothing about the cost implications for healthcare providers of either recontacting, or not recontacting, patients and patient groups. Nor is there insight in the literature on the views and the potential roles of charitable or third sector organisations in relation to recontacting. Questions about the threshold of information that triggers a recontact is likely to become more complex as knowledge about gene–gene interactions becomes available. Overall, then, more research is needed to support the evolution of this debate. At this juncture, it is unclear if recontacting is to remain an issue for individual professionals as they consider information relevant to a specific patient or patient group, or is to be integrated into a system of roles and responsibilities of genetics services as they manage patient information. My sense is that it will become the latter; in which case research of the kind outlined above is needed.

## References

[CR2] Appelbaum PS, Parens E, Berger SM, Chung WK, Burke W (2020). Is there a duty to reinterpret genetic data? The ethical dimensions. Genet Med.

[CR3] Beunders G, Dekker M, Haver O, Meijers-Heijboer HJ, Henneman L (2018). Recontacting in light of new genetic diagnostic techniques for patients with intellectual disability: Feasibility and parental perspectives. Eur J Med Genet.

[CR4] Bombard Y, Mighton C (2019). Recontacting clinical genetics patients with reclassified results: equity and policy challenges. Eur J Hum Genet.

[CR5] Carrieri D, Lucassen AM, Clarke AJ, Dheensa S, Doheny S, Turnpenny PD, Kelly SE (2016). Recontact in clinical practice: a survey of clinical genetics services in the United Kingdom. Genet Med.

[CR6] Carrieri D, Dheensa S, Doheny S, Clarke A, Turnpenny P, Lucassen A, Kelly S (2017). Recontacting in clinical practice: an investigation of the views of healthcare professionals and clinical scientists in the United Kingdom. Eur J Hum Genet.

[CR7] Carrieri D, Dheensa S, Doheny S, Clarke A, Turnpenny PD, Lucassen A, Kelly S (2017). Recontacting in clinical practice: the views and expectations of patients in the United Kingdom. Eur J Hum Genet.

[CR8] Carrieri D, Howard HC, Benjamin C, Clarke AJ, Dheensa S, Doheny S, Hawkins N, Halbersma-Konings TF, Jackson L, Kayserili H, Kelly SE, Lucassen AM, Mendes A, Rial-Sebbag E, Stefansdottir V, Turnpenny PD, van El CG, van Langen IM, Cornel MC, Forzano F (2019). Recontacting patients in clinical genetics services: recommendations of the European Society of Human Genetics. Eur J Hum Genet.

[CR9] David KL, Best RG, Manace Brenman L, Bush L, Deignan JL, Flannery D, Hoffman JD, Holm I, Miller DT, O’Leary J, Pyeritz RE, on behalf of the ACMG Social Ethical Legal Issues Committee (2019) Patient re-contact after revision of genomic test results: points to consider—a statement of the American College of Medical Genetics and Genomics (ACMG). Genet Med 21(4):769–771 10.1038/s41436-018-0391-z 30578420

[CR10] de Paor A (2018). Genetic risks and doctors’ disclosure obligations—revisiting the duty of confidentiality. Eur J Health Law.

[CR11] Dheensa S, Carrieri D, Kelly S, Clarke A, Doheny S, Turnpenny P, Lucassen A (2017). A 'joint venture' model of recontacting in clinical genomics: challenges for responsible implementation. Eur J Med Genet.

[CR12] Doheny S, Clarke A, Carrieri D, Dheensa S, Hawkins N, Lucassen A, Turnpenny P, Kelly S (2018). Dimensions of responsibility in medical genetics: Exploring the complexity of the duty to recontact. New Genet Soc.

[CR13] Dove ES, Chico V, Fay M, Laurie G, Lucassen AM, Postan E (2019). Familial genetic risks: how can we better navigate patient confidentiality and appropriate risk disclosure to relatives?. J Med Ethics.

[CR14] El Mecky J, Johansson L, Plantinga M, Fenwick A, Lucassen A, Dijkhuizen T, van der Hout A, van Lyle K, Langen I (2019). Reinterpretation, reclassification, and its downstream effects: challenges for clinical laboratory geneticists. BMC Med Genomics.

[CR15] Fitzpatrick JL, Hahn C, Costa T, Huggins MJ (1999). The duty to recontact: attitudes of genetics service providers. Am J Hum Genet.

[CR16] Hirschhorn K, Fleisher LD, Godmilow L, Howell RR, Lebel RR, McCabe ERB (1999). Duty to re-contact policy statement: social ethical and legal issues committee of the American College of Medical Geneticists. Genet Med.

[CR17] Joint Committee on Genomics in Medicine (2019). Consent and confidentiality in genomic medicine: Guidance on the use of genetic and genomic information in the clinic.

[CR18] Mitchell C, Ploem C, Retel V, Gevers S, Hennekam R (2020). Experts reflecting on the duty to recontact patients and research participants; why professionals should take the lead in developing guidelines. Eur J Med Genet.

[CR19] Otten E, Plantinga M, Birnie E, Verkerk MA, Lucassen AM, Ranchor AV, Van Langen IM (2015). Is there a duty to recontact in light of new genetic technologies? A systematic review of the literature. Genet Med.

[CR20] Ploem C, Mitchell C, van Harten W, Gevers S (2018). A duty to recontact in the context of genetics: futuristic or realistic?. Eur J Health Law.

[CR22] Richards CS, Bale S, Bellissimo DB, Das S, Grody WW, Hegde MR, Lyon E, Ward BE (2007). Molecular Subcommittee of the ACMG Laboratory Quality Assurance Committee. ACMG recommendations for standards for interpretation and reporting of sequence variations. Rev Genet Med.

[CR23] Rose N (2007). The politics of life itself: biomedicine, power and subjectivity in the twenty-first century.

[CR24] Sirchia F, Carrieri D, Dheensa S, Benjamin C, Kayserili H, Cordier C, van El CG, Turnpenny PD, Melegh B, Mendes Á, Halbersma-Konings TF, van Langen IM, Lucassen AM, Clarke AJ, Forzano F, Kelly SE (2018). Recontacting or not recontacting? A survey of current practices in clinical genetics centres in Europe. Eur J Hum Genet.

[CR25] Tudor Hart J (1971). The inverse care law. Lancet.

[CR26] Vears DF, Niemiec E, Howard HC, Borry P (2018). Analysis of VUS reporting, variant reinterpretation and recontact policies in clinical genomic sequencing consent forms. Eur J Hum Genet.

[CR27] Vears DF, Sénécal K, Clarke AJ, Jackson L, Laberge AM, Lovrecic L, Piton A, Li Van Gassen K, Yntema HG, Knoppers BM, Borry P (2018). Points to consider for laboratories reporting results from diagnostic genomic sequencing. Eur J Hum Genet (EJHG).

